# Supplementary material for: Using Wastewater Surveillance to Compare COVID-19 Outbreaks during the Easter Holidays over a 2-Year Period in Cape Town, South Africa
Source: Viruses. 2023 Jan 5;15(1):162. doi: 10.3390/v15010162 (PMC9863979; doi:10.3390/v15010162)
Supplement: Supplementary file 1 [file viruses-15-00162-s001.zip › viruses-2115494-SI.pdf]

Supplementary Material

Using wastewater surveillance to compare COVID-19 outbreaks during the Easter holidays over a 2-year period in Cape Town, South Africa

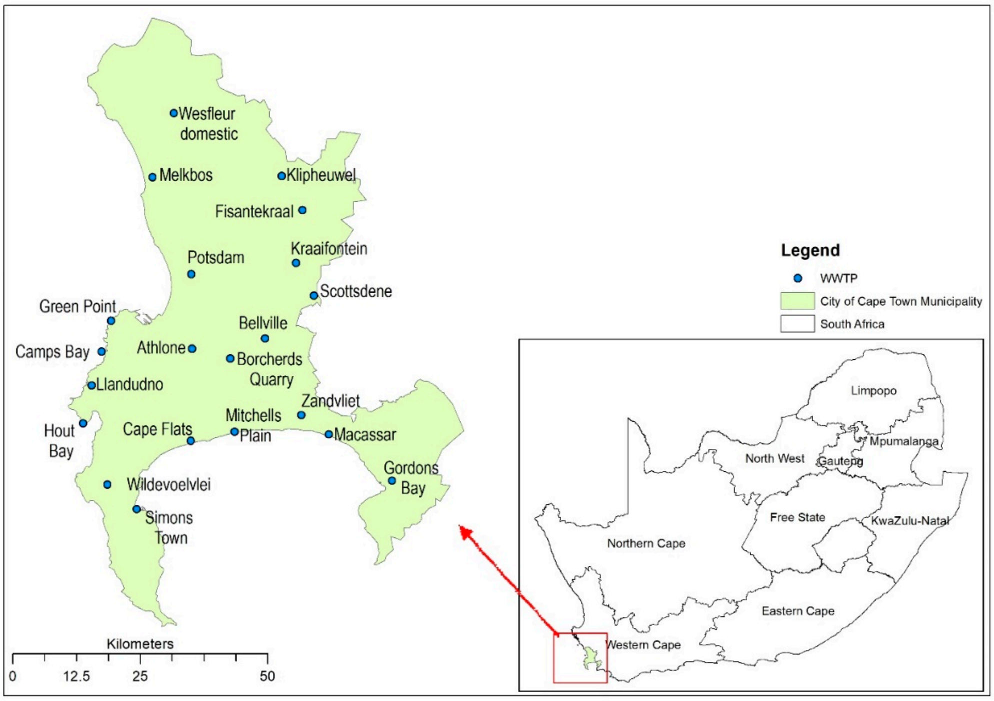

Figure S1. Distribution of the 21 wastewater treatment plants (WWTPs) in the City of Cape Town municipality

Table S1. Characteristics of the wastewater treatment plants in the City of Cape Town

**WWTP**	**Population served**	**Capacity (Ml/d)**
Athlone	463,733	105
Bellville	234,699	54.6
Borcherds Quarry	249,643	35
Camps Bay	6019	5.5
Cape Flats	1,125,632	200
Fisantekraal	16,356	24
Gordons Bay	19,982	3.1
Green Point	65,871	40
Hout Bay	44,108	9.8
Klipheuwel	5235	0.07
Kraaifontein	88,154	17.5
Llandudno	621	0.28
Macassar	267,288	38
Melkbosstrand	17,561	5.4
Mitchells Plain	445,302	45
Potsdam	238,479	47
Scottsdene	148,921	12.5
Simons Town	6997	2.5
Wesfleur Domestic	92,575	8
Wildevoelvlei	75,702	14
Zandvliet	1,090,368	72

Table S2. Thermal cycling conditions and details for N1 and N2 probes used in the study

Organism	Target	Assay name	Target	Part Number	Reference
SARS-CoV-2	Nucleocapsid protein	2019-nCoV CDC	N1 primer/probe	RV202001 RV202015	[1]
			N2 primer/probe	RV202002 RV202016	

Reference

1. Johnson, R.; Muller, C. J. F.; Ghoor, S.; Louw, J.; Archer, E.; Surujlal-Naicker, S.; Berkowitz, N.; Volschenk, M.; Bröcker, L. H. L.; Wolfaardt, G.; Van der Walt, M.; Mutshembele, A. M.; Malema, S.; Gelderblom, H. C.; Mdhluli, M.; Gray, G.; Mathee, A.; Street, R. Qualitative and quantitative detection of SARS-CoV-2 RNA in untreated wastewater in Western Cape Province, South Africa. *S. Afr. Med. J.* **2021**.*111*(3), 198. https://doi.org/10.7196/SAMJ.2021.v111i3.15154
